# Anti-thyroid peroxidase antibody and thyroid cysts among the general Japanese population: a cross-sectional study

**DOI:** 10.1186/s12199-020-00844-x

**Published:** 2020-02-21

**Authors:** Yuji Shimizu, Yuko Nabeshima-Kimura, Shin-Ya Kawashiri, Yuko Noguchi, Yasuhiro Nagata, Takahiro Maeda, Naomi Hayashida

**Affiliations:** 1 Department of Community Medicine, Nagasaki University Graduate School of Biomedical Sciences, Sakamoto 1-12-4, Nagasaki, 852-8523 Japan; 2 Department of Cardiovascular Disease Prevention, Osaka Center for Cancer and Cardiovascular Disease Prevention, Osaka, Japan; 3 Department of Radiation Health Management, Fukushima Medical University, Fukushima, Japan; 4 Center for Comprehensive Community Care Education, Nagasaki University Graduate School of Biomedical Sciences, Nagasaki, Japan; 5 Department of General Medicine, Nagasaki University Graduate School of Biomedical Sciences, Nagasaki, Japan; 6 Division of Promotion of Collaborative Research on Radiation and Environment Health Effects, Atomic Bomb Disease Institute, Nagasaki University, Nagasaki, Japan

**Keywords:** Anti-thyroid peroxidase antibody, Cross-sectional study, Normal thyroid function, Thyroid cysts, Thyroid hormone

## Abstract

**Background:**

Anti-thyroid peroxidase antibody (TPO-Ab) has been shown to cause autoimmune thyroiditis by inducing a deleterious influence on thyroid hormone synthesis. Further, thyroglobulin, which has an important role in thyroid hormone synthesis, is reported to be high in the fluid from thyroid cysts. Therefore, TPO-Ab could be associated with the presence of thyroid cyst, partly by affecting the activity of thyroid hormone synthesis.

**Methods:**

To investigate the association between TPO-Ab and thyroid cysts, we conducted a cross-sectional study of 1432 Japanese with normal thyroid function [i.e., normal range of free triiodothyronine (free T3) and free thyroxine (free T4)] between the ages of 40 and 74 years, who participated in an annual health check-up.

**Results:**

In men, the statistical power did not reach a statistical significance value. Additionally, subjects with TPO-Ab showed lower odds ratios (ORs) of thyroid cysts than those without TPO-Ab. In women, subjects with TPO-Ab showed significantly lower ORs of thyroid cysts than those without TPO-Ab. The fully adjusted ORs were 0.68 (0.40, 1.18) for men and 0.40 (0.27, 0.60) for women. When evaluating the association between logarithmic values of TPO-Ab titer and thyroid cysts in both men and women, a notable inverse correlation was observed. The fully adjusted ORs were 0.68 (0.50, 0.92) for men and 0.68 (0.57, 0.81) for women.

**Conclusion:**

TPO-Ab titer revealed to be inversely associated with thyroid cysts among Japanese with normal thyroid function. The presence of a thyroid cyst could indicate a lower risk of having TPO-Ab among the general population with normal thyroid function.

## Background

Anti-thyroid peroxidase antibody (TPO-Ab) inhibits thyroid hormone [triiodothyronine (T3) and thyroxine (T4)] synthesis because thyroid peroxidase takes an important role in synthesizing thyroid hormone [1]. Therefore, subjects with TPO-Ab should display a reduced rate of thyroid hormone synthesis, even among subjects with normal thyroid function. To sustain a normal thyroid function in the presence of an inefficient T3 and T4 production, subjects with TPO-Ab may display an overstimulated synthesis of thyroid hormones compared with those without TPO-Ab. This mechanism speculates that even among subjects with normal thyroid function, those with TPO-Ab will display significantly higher levels of thyroid-stimulating hormone (TSH) when compared with those without TPO-Ab.

Additionally, the previous study reported noticeably increased thyroglobulin in the fluid from thyroid cysts [2]. Since thyroglobulin plays a crucial role in synthesizing thyroid hormones [3], the presence of thyroglobulin-rich cysts could have a beneficial influence on thyroid hormone synthesis. Therefore, elevated synthesis of thyroid hormones may cause an increase in thyroglobulin consumption, which could result in a lowered presence of thyroid cysts.

According to the aforementioned mechanism, the presence of TPO-Ab could be inversely associated with thyroid cysts among subjects with normal thyroid function and could be indicative of the inefficient production of thyroid hormones. Since thyroid cysts are generally considered to lack clinical significance, the potential association between TPO-Ab and thyroid cysts has never been investigated.

To evaluate this association, we conducted a cross-sectional study of 1432 Japanese subjects with normal thyroid function (normal range of free T3 and free T4) aged 40–74 years who participated in an annual health check-up in 2014.

## Material and methods

### Study population

The study population comprised 1883 Japanese people between the ages of 40 and 74 years from the Saza town in the western part of Japan who underwent an annual medical check-up in 2014, as recommended by the Japanese government.

To avoid the influence of thyroid disease, subjects with a history of thyroid disease (*n* = 60), without thyroid function data such as TSH, free T3, and free T4 (*n* = 17), and subjects with an abnormal T3 (normal range 2.1–4.1 pg/mL) and T4 (normal range 1.0–1.7 ng/dL) range were excluded (*n* = 77).

Additionally, subjects without body mass index (BMI) data (*n* = 1), blood pressure data (*n* = 1), TPO-Ab data (*n* = 294), and women without menopause data (*n* = 1) were excluded. The remaining subjects, comprising 1432 with a mean age of 60.9 years (standard deviation (SD) 9.0; range 40–74) were enrolled in the study.

### Data collection and laboratory measurements

A trained interviewer obtained information on clinical characteristics. Bodyweight and height were measured with an automatic body composition analyzer (BF-220; Tanita, Tokyo, Japan) and BMI (kg/m^2^) was calculated. Systolic blood pressure (SBP) was recorded at rest.

A fasting blood sample was collected. TSH, free T3, free T4, and TPO-Ab were measured by standard procedures at the LSI Medience Corporation (Tokyo, Japan). HbA1c, triglycerides (TG), and high-density lipoprotein cholesterol (HDLc) were also measured using standard procedures at SRL, Inc. (Tokyo, Japan).

Detecting thyroid cysts are identified by experienced technicians using a LOGIQ Book XP with a 10-MHz transducer (GE Healthcare, Milwaukee, WI, USA). A thyroid cyst (maximum diameter ≥ 2.0 mm) without a solid component was defined as a thyroid cyst for this study. The positive status of TPO-Ab (+) was defined at and above 16 IU/mL.

### Statistical analysis

Characteristics of the study population were expressed as mean ± SD except for anti-hypertensive medication use, menopause, TPO-Ab, and TSH. The status of anti-hypertensive medication use and menopause was expressed as a percent value. Since TPO-Ab and TSH showed a skewed distribution, the characteristics of this study population were expressed as median [the first quartile, the third quartile]. The differences among free T3, free T4, and TSH regarding the status of TPO-Ab were calculated. Significant differences by the status of TPO-Ab were evaluated using analysis of variance (ANOVA).

Logistic regression models were used to calculate odds ratios (ORs) and 95% confidence intervals (CIs) to determine the association between TPO-Ab and thyroid cysts. Three adjustment models were used. The first model was adjusted only for sex and age (model 1); the second model (model 2) further included the potential confounding factors that were directly associated with thyroid function, namely TSH (μIU/mL), free T3 (pg/mL), and free T4 (ng/dL). The last model (model 3) was further adjusted for potential confounding factors that were indirectly associated with thyroid function; such as, BMI (kg/m^2^), SBP (mmHg), anti-hypertensive medication use (yes/no), HbA1c (%), TG (mg/dL), HDLc (mg/dL), and for women menopause (yes/no). We also stratified the participant by the status of gender.

All statistical analysis was performed with the SAS system for Windows (version 9.4: SAS Inc., Cary, NC, USA). Values of *p* < 0.05 were regarded as statistically significant.

## Results

Table 1 shows the characteristics of the study population. Among the study population, 146 (27.4%) men and 323 (35.9%) women had thyroid cysts.

**Table 1:** Characteristics of the study population

	Total	Men	Women
No. of participants	1432	532	900
No. of case (%)	469 (32.8)	146 (27.4)	323 (35.9)
TPO-Ab, (< 16) IU/mL	9 [7, 13]^*1^	9 [7, 12]^*1^	9 [7, 13]^*1^
Age, year	60.9 ± 9.0	62.4 ± 8.7	59.9 ± 9.1
TSH, (0.39–4.01) μIU/mL	1.59 [1.11, 2.28]^*1^	1.53 [1.07, 2.16]^*1^	1.61 [1.13, 2.33]^*1^
Free T3, (2.1–4.1) pg/mL	3.2 ± 0.3	3.3 ± 0.3	3.1 ± 0.3
Free T4, (1.0–1.7) ng/dL	1.2 ± 0.2	1.3 ± 0.2	1.2 ± 0.1
BMI, kg/m^2^	22.8 ± 3.4	23.6 ± 3.1	22.4 ± 3.5
SBP, mmHg	125 ± 17	129 ± 14	123 ± 18
Anti-hypertensive medication, %	30.9	38.7	26.3
HbA1c, %	5.6 ± 0.6	5.7 ± 0.7	5.6 ± 0.6
TG, mg/dL	105 ± 73	123 ± 91	95 ± 56
HDLc, mg/dL	61 ± 15	54 ± 14	64 ± 14
Menopause, %	50.3	–	80.0

*TPO-Ab* anti-thyroid peroxidase antibody, *TSH* thyroid-stimulating hormone, *free T3* free triiodothyronine, *free T4* free thyroxine, *BMI* body mass index, *SBP* systolic blood pressure, *TG* triglycerides, *HDLc* high-density lipoprotein cholesterol

No. of case is the number of participants with thyroid cyst. Values are mean ± standard deviation

*^1^Values are median [the first quartile, third quartile]. Normal range of measurements are ( )

The values of the thyroid-related hormone by TPO-Ab status are shown in Table 2. TPO-Ab (+) showed significantly higher values of TSH than TPO-Ab (–). However, no significant differences between TPO-Ab (+) and TPO-Ab (–) were observed for free T3 and free T4.

**Table 2:** Thyroid-related hormone by anti-thyroid peroxidase antibody (TPO-Ab)

	Anti-thyroid peroxidase antibody (TPO-Ab)		*p*
	−	+	
Total			
No. of participants	1165	267	
TSH, μIU/mL	1.53 [1.09, 2.20]^*1^	1.80 [1.20, 2.66]^*1^	< 0.001^*2^
Free T3, pg/mL	3.2 ± 0.3	3.2 ± 0.3	0.990
Free T4, ng/dL	1.2 ± 0.2	1.3 ± 0.2	0.395
Men			
No. of participants	441	91	
TSH, μIU/mL	1.49 [1.06, 2.05]^*1^	1.75 [1.16, 2.60]^*1^	0.026^*2^
Free T3, pg/mL	3.3 ± 0.3	3.3 ± 0.3	0.997
Free T4, ng/dL	1.3 ± 0.2	1.3 ± 0.2	0.912
Women			
No. of participants	724	176	
TSH, μIU/mL	1.57 [1.11, 2.28]^*1^	1.88 [1.25, 2.74]^*1^	0.005^*2^
Free T3, pg/mL	3.1 ± 0.3	3.1 ± 0.3	0.657
Free T4, ng/dL	1.2 ± 0.1	1.2 ± 0.1	0.148

*TSH* thyroid-stimulating hormone, *free T3* free triiodothyronine, *free T4* free thyroxine

Values are mean ± standard deviation

*^1^Values are median [the first quartile, third quartile]. *^2^Logarithmic transformation was used for evaluating *p*

Table 3 shows ORs and 95% CIs of thyroid cysts regarding the status of TPO-Ab for total subjects. Independent of known confounding factors, compared with the reference group of TPO-Ab (–), TPO-Ab (+) showed significantly lower ORs for thyroid cysts. Furthermore, the logarithmic values of TPO-Ab titer also were notably inversely associated with thyroid cysts.

**Table 3:** Odds ratios (ORs) and 95% confidence intervals (CIs) for thyroid cyst in relation to anti-thyroid peroxidase antibody (TPO-Ab)

	Anti-thyroid peroxidase antibody		*p*	TPO-Ab (logarithmic values)
	−	+		
No. of participants	1165	267		
No. of case	411 (35.3)	58 (21.7)		
Model 1	1	0.47 (0.34, 0.65)	< 0.001	0.67 (0.58, 0.78)
Model 2	1	0.47 (0.34, 0.65)	< 0.001	0.67 (0.58, 0.78)
Model 3	1	0.47 (0.34, 0.65)	< 0.001	0.67 (0.58, 0.78)

Model 1: adjusted for sex and age. Model 2: + TSH, free T3, free T4. Model 3: + BMI, SBP, anti-hypertensive medication, TG, HDLc, HbA1c, and for women menopause

Sex-specific ORs and 95% CIs of thyroid cysts concerning the status of TPO-Ab are shown in Table 4. For men, even the statistical power could not reach significant value, compared with the reference group of TPO-Ab (–), TPO-Ab (+) showed lower ORs of thyroid cyst. For women, TPO-Ab (+) showed significantly lower ORs of thyroid cyst than TPO-Ab (–). When we evaluate the association between logarithmic values of TPO-Ab and thyroid cyst, for both men and women, significantly inversely associations are observed.

**Table 4:** Sex-specific odds ratios (ORs) and 95% confidence intervals (CIs) for thyroid cyst in relation to anti-thyroid peroxidase antibody (TPO-Ab)

	Anti-thyroid peroxidase antibody		p	TPO -Ab (logarithmic values)
	-	+		
Men				
No of participants	441	91		
No. of case	125 (28.3)	21 (23.1)		
Model 1	1	0.69 (0.40, 1.18)	0.174	0.69 (0.51, 0.94)
Model 2	1	0.68 (0.40, 1,17)	0.160	0.67 (0.49, 0.92)
Model 3	1	0.68 (0.40, 1.18)	0.169	0.68 (0.50, 0.92)
Women				
No of participants	724	176		
No. of case	286 (39.5)	37 (21.0)		
Model 1	1	0.39 (0.26, 0.58)	<0.001	0.67 (0.56, 0.79)
Model 2	1	0.40 (0.27, 0.59)	<0.001	0.67 (0.56, 0.80)
Model 3	1	0.40 (0.27, 0.60)	<0.001	0.68 (0.57, 0.81)

Model 1: adjusted for age. Model 2: + TSH, free T3, free T4. Model 3: + BMI, SBP, anti-hypertensive medication, TG, HDLc, HbA1c, and for women menopause

## Discussion

The major findings of the present study were that in both men and women, a higher titer of TPO-Ab was associated with the absence of thyroid cysts among the general population with normal thyroid function.

Even in the presence of anti-thyroglobulin-antibodies, which decreases the overall thyroglobulin level, significant increases in thyroglobulin levels were reported in the fluid from thyroid cysts [2]. Since thyroglobulin plays an important role in thyroid hormone (T3 and T4) synthesis [3], subjects with thyroglobulin rich thyroid cyst might have a beneficial effect on synthesizing thyroid hormone (T3 and T4). School years are a crucial period of growth, and thyroid hormone is important for physical development [4]. The number of thyroid cysts among school-aged children has been found to increase with age [5]. Such findings also support the hypothesis that thyroid cysts may have a beneficial effect on thyroid hormone synthesis.

Thyroid peroxidase plays an important role in thyroid hormone (T3 and T4) synthesis [1]. Therefore, thyroid peroxidase inhibition caused by TPO-Ab may result in reduced synthesis of thyroid hormones. Nonetheless, we observed no significant difference in the values of thyroid hormones (free T3 and free T4) between TPO-Ab (+) and TPO-Ab (–). Inefficient synthesis of thyroid hormones among subjects with TPO-Ab may be responsible for these results. Inefficient thyroid hormone synthesis can increase the consumption rate of thyroglobulin, which reduces the chance of forming cysts in the thyroid. In fact, in this study, TPO-Ab (+) showed significantly higher values of TSH, which stimulates thyroid hormone production, than that of TPO-Ab (–).

Additionally, thyroid peroxidase is found in follicle cells of the thyroid [6]. Therefore, activating TPO-Ab-related inflammatory response may result in the degradation of thyroid follicles. Given the histopathological characteristics of this inflammatory response could result in chronic thyroiditis.

It is well-documented that TPO-Ab is a known risk factor for autoimmune thyroid disease. Even subjects with TPO-Ab (+), showing normal thyroid function, possess a risk of abnormal thyroid function in the future. In this study, we found a significant inverse association between TPO-Ab and thyroid cysts among subjects with normal thyroid function, possibly indicating latent damage of thyroid function. If so, the prevalence of cysts among TPO-Ab positive subjects could be an efficient diagnostic tool in evaluating TPO-Ab-related latent damage to the thyroid. Further investigation is necessary.

The prevalence of thyroid nodules among the general population has also been well-documented [7]. Nevertheless, to our knowledge, no previous studies have reported the prevalence of thyroid cysts among the general Japanese population in those between 40 and 74 years old. Although the present study population is not adequately representative of the entire Japanese general population, our study clarifies the prevalence of thyroid cysts among the general population to some degree (27.4% for men and 35.9% for women). This is a valuable contribution to this work.

The potential limitations of this study do warrant consideration. First, we evaluated the existence of a thyroid cyst on the parameters whether it was present or not. However, the number and size of a given cyst could be an important factor. Further investigation with this data is necessary. Due to the limited amount of blood samples, we could not evaluate the influence of anti-thyroglobulin antibodies, which may act as a strong confounding factor. Further investigation with data of the anti-thyroglobulin antibody is necessary. Also, this was a cross-sectional study where a causal relationship could not be established.

## Conclusion

In conclusion, in subjects with normal thyroid function, a higher TPO-Ab titer is associated with the absence of thyroid cysts. Even with further investigation, the present findings could be an efficient diagnostic tool in developing an evaluation method for the magnitude of latent thyroid damage among the general population with normal thyroid function.

## Data Availability

The datasets generated during and/or analyzed during the current study are not publicly available due to ethical consideration but are available from the corresponding author on reasonable request.

## Abbreviations

ANOVA: Analysis of variance
BMI: Body mass index
CIs: Confidence intervals
HDLc: High-density lipoprotein cholesterol
ORs: Odds ratios
SBP: Systolic blood pressure
SD: Standard deviation
T3: Triiodothyronine
T4: Thyroxine
TG: Triglycerides
TPO-Ab: Anti-thyroid peroxidase antibody
TSH: Thyroid-stimulating hormone
